# ^137^Cs and ^40^K in gray seals *Halichoerus grypus* in the southern Baltic Sea

**DOI:** 10.1007/s11356-019-05145-7

**Published:** 2019-04-24

**Authors:** Michał Saniewski, Tamara Zalewska, Maria Suplińska, Lucyna Falkowska, Agnieszka Grajewska, Iga Nehring, Dominika Saniewska, Marta Staniszewska, Iwona Pawliczka

**Affiliations:** 1 Institute of Meteorology and Water Management - National Research Institute, Waszyngtona 42, 81-342 Gdynia, Poland; 2 Central Laboratory for Radiological Protection, Konwaliowa 7, 03-194 Warsaw, Poland; 3 Institute of Oceanography, University of Gdańsk, Al. Marszałka Piłsudskiego 46, 81-378 Gdynia, Poland

**Keywords:** Radionuclides, Gray seals, Baltic Sea

## Abstract

This study presents levels of ^137^Cs and ^40^K concentrations in the placentas of seals gathered in the period 2007–2015. The mean activity of ^137^Cs and ^40^K was 5.49 Bq kg^−1^w.w. and 136.6 Bq kg^−1^ ww respectively. Statistically significant correlation was observed between the ^137^Cs activities in placenta and in herring—the staple food for seals. The concentrations of ^137^Cs and ^40^K were also determined in other tissues (muscle, liver, lung, and brain) of wild seals. The concentrations of ^137^Cs were from 2.59 Bq^−1^ ww (lungs) to 24.3 Bq kg^−1^ ww (muscles). The transfer factor values for ^137^Cs (seal tissue/fish) ranged from 0.89 to 2.42 in the case of the placentas and from 1.35 to 8.17 in the case of the muscle. For adults seal, the effective dose from ^137^Cs was 2.98 nGy h^−1^. The mean external radiation dose to pup was 0.77 nGy h^−1^ from ^137^Cs and 6.69 nGy h^−1^ from ^40^K.

## Introduction

The Baltic Sea, being a largely enclosed area in terms of its connection with ocean waters, is much more sensitive to pollution than other seas. At present, despite the lack of significant sources of ^137^Cs, its concentrations still remain significantly higher compared to other marine areas, which is a result of significant amounts of radionuclides introduced in the past (IAEA 2005). The major sources of ^137^Cs input into the Baltic Sea were the Chernobyl Nuclear Power Plant accident in 1986 and the atmospheric tests of nuclear weapons carried out during the late 1950s and early 1960s. The total deposition was assessed to amount to 800 TBq after the nuclear weapons tests and 4700 TBq following the Chernobyl accident. In the period 2005–2011, the average annual load of ^137^Cs from the Vistula River to the Gulf of Gdańsk was 406 GBq and from atmospheric fallout was 18.1 GBq (Saniewski and Zalewska 2016). Long-term observations of the changes in concentrations of ^137^Cs in Baltic waters show that from 1991, the continuous decrease is observed (Zalewska and Suplińska 2013). Although the Vistula water is still sources of ^137^Cs to the Gulf of Gdańsk, the input of ^137^Cs is compensated by processes such as radioactive decay of ^137^Cs, sediment deposition, and in minor extent bioaccumulation in flora and fauna organisms like macrophytobenthic plants and fish. In the period 2005–2011, the shares of these processes in decrease of ^137^Cs activity in the Gulf of Gdańsk were 355 GBq year^−1^ (61.0%) in the case of sediment deposition, radioactive decay 227 GBq year^−1^ (38.95%), and bioaccumulation in macrophytobenthic plants and fishes 0.3 GBq year^−1^ (0.05%). However, the activity of this isotope in the Baltic Sea is still higher than 30 years ago, before the failure at the Chernobyl Nuclear Power Plant. Therefore, studies have been undertaken to determine the activity of ^137^Cs and ^40^K in various tissues of the gray seal (*Halichoerus grypus*), especially in the placentas of seals from breeding, as potential sources of external radiation for pups. The available data regarding seals is very limited, as shown by the comparatively small number of publications presenting the actual concentrations of ^137^Cs in the tissues of the seal from the Baltic Sea (Ciesielski et al. 2015; Saremi et al. 2018) and in other areas like Svalbard, Barents Sea, Greenland Sea, and Arviat area of Canada (e.g., Carroll et al. 2002; Andersen et al. 2006; Chen et al. 2017).

The gray seal is one of the four marine mammal species found in the Baltic Sea whose numbers significantly dropped in the first half of the twentieth century. As a result of extermination by fisherman, bycatch and the effects of environmental pollution with hazardous substances (DDT, PCB) which caused serious damage to female reproductive organs, gray seal populations dropped almost 4 times in the period from 1940 to 1980. At present, the species is protected in the whole Baltic Sea area. In addition, the gray seal is covered by a program intended to recover (reintroduce) its population in the Baltic Sea, a task undertaken by the Marine Station of the Institute of Oceanography at the University of Gdańsk (MSIOUG). Reintroduce program enable possible to conduct research to determine the levels of radioactive ^137^Cs in seal placentas, which would be difficult to carry out in natural conditions because of the fact that seals eat the placenta immediately after birth. That in turn made it possible to identify the potential exposure of young seals to ionizing radiation connected with ^137^Cs and compare it with level arising from the activity of the natural isotope of ^40^K. In order to check how ^137^Cs concentrations in fish, the main food of seals can affect the levels observed in the body; ^137^Cs analyses were carried out in herring caught in the southern Baltic during the same period. As an additional element, the concentrations of ^137^Cs and ^40^K were also determined in other tissue: muscle, liver, lung, and brain of seal found along the Polish coast in the period 2013–2015.

The aim of the study was to check whether ^137^Cs levels observed in various seal tissues, with particular reference to the placenta, whose main source is ^137^Cs in the diet (herring), may pose a significant threat to fetuses during pregnancy.

## Materials and methods

### Materials characteristics

**Fig. 1:**
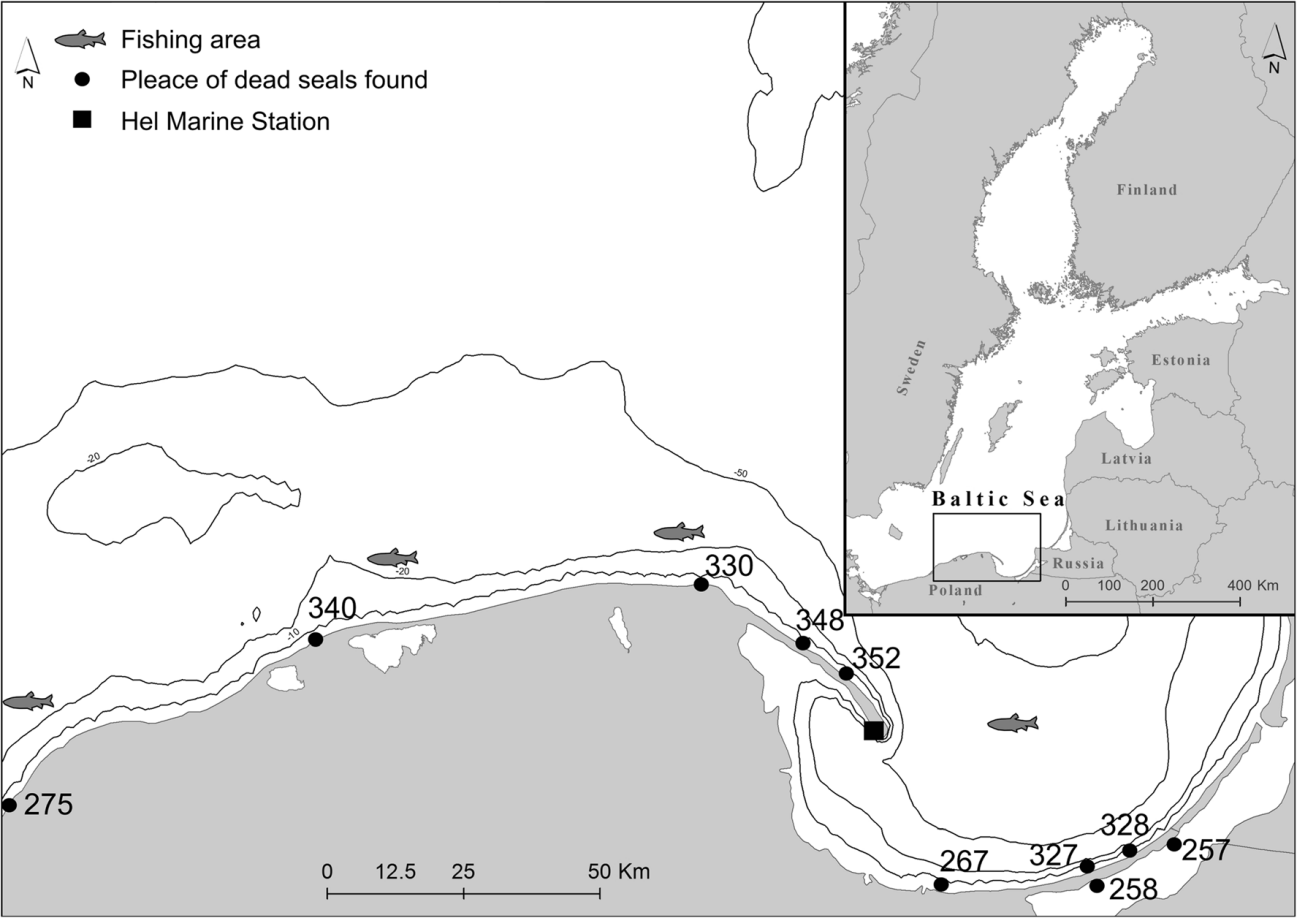
Localization of sampling stations

Activity concentrations of ^137^Cs and ^40^K were determined in seal placenta samples collected in the years 2007–2015 from the Marine Station of the Institute of Oceanography at the University of Gdańsk (MSIOUG). The breeding center is permanently inhabited by a group of 6 seals (4 females and 2 males). The females in the group were born in the wild. The oldest female (1) was found off the coast of Sweden as a pup. The other 3 females (2, 3, 4) come from the Estonian island of Allirahu, where after 3 weeks of being nursed by their mothers, they were caught and transported to the Hel Marine Station where they have stayed since 1998 (see Nehring et al. 2017, for more details). Up to 4 seal pups are born every year at the MSIOUG. After the period of being fed by the mothers (about 3 weeks), all pups are released into the natural environment in order to rebuild the seal population in the southern Baltic and continuation of migration research. Samples of 24 seal placenta (23 following birth and one after a miscarriage which happened 2 months before the due date) were collected at the Marine Station run by the Institute of Oceanography of the University of Gdansk (Fig. 1, Table 1). In addition, in two placenta samples, a sum of concentrations of plutonium isotopes was assayed—^239 + 240^Pu.

**Table 1:** Radionuclide activities in the placenta of the gray seal from the Hel Marine Station

Female’s number	Puppy weight (kg)	Date of birth	Weight of the bearing	^137^Cs (Bq kg ww)	TF (^137^Cs)	^40^K (Bq kg ww)	TF (^40^K)	^239 + 240^Pu (mBq kg ww)
2	–	28.02.2007	1.48	10.48 ± 0.58	1.95	123.4 ± 31.2	1.10	
3	–	27.02.2007	1.38	5.77 ± 0.57	1.07	154.0 ± 35.7	1.37	5.00 ± 3.46
1	15.05	1.03.2008	1.25	12.32 ± 0.97	2.42	178.8 ± 45.4	1.47	3.31 ± 2.48
3	17.7	1.03.2008	1.78	5.32 ± 0.67	1.04	125.5 ± 36.5	1.03	
2	14.9	5.03.2008	1.39	5.75 ± 0.65	1.13	< 46		
1	16	4.03.2009	1.7	4.20 ± 0.61	0.85	124.9 ± 32.2	0.96	
2	15.7	3.03.2009	1.4	6.97 ± 1.42	1.41	273.2 ± 84.3	2.11	
2	15.8	3.10.2010	1.5	4.18 ± 0.70	0.89	148.9 ± 43.0	1.12	
3	19.5	2.3.2010	2.6	6.25 ± 0.85	1.34	106.8 ± 46.0	0.81	
1	14.1	8.3.2010	1.3	4.95 ± 0.80	1.06	114.1 ± 40.5	0.86	
3	15.8	6.3.2011	1.8	4.45 ± 0.56	1.18	131.6 ± 33.7	1.23	
4	13.3	10.3.2011	1.1	4.89 ± 0.68	1.30	101.4 ± 40.7	0.95	
2	16.2	11.3.2011	1.48	4.57 ± 0.65	1.21	114.8 ± 41.6	1.07	
3	16.7	9.3.2012	1.5	3.80 ± 0.64	0.98	120.9 ± 34.6	0.97	
1	16.4	6.3.2012	1.44	3.54 ± 0.58	0.91	107.2 ± 34.1	0.86	
2	15.5	7.3.2012	1.37	7.44 ± 1.01	1.92	170.1 ± 53.8	1.37	
3	15.3	3.3.2013	2.0	5.62 ± 0 .75	1.64	143.5 ± 48.6	1.26	
1	15.9	5.3.2013	1.7	4.55 ± 0.66	1.33	144.9 ± 41.9	1.27	
1	–	13.12.2013 (still birth)	0.62	5.27 ± 0.51	1.54	82.7 ± 28.4	0.73	
2	17	10.3.2014	1.4	4.77 ± 0.61	1.51	162.7 ± 36.8	1.48	
3	16.5	8.3.2014	1.8	3.63 ± 0.58	1.15	147.3 ± 3 9.9	1.34	
1	–	23.2.2015	–	3.55 ± 0.61	1.20	112.7 ± 38.9	1.01	
4	–	1.3.2015	–	4.69 ± 0.73	1.58	105.0 ± 49.0	0.94	
3	–	6.3.2015	–	4.79 ± 0.68	1.61	147.9 ± 40.0	1.32	

Analysis to determine ^137^Cs and ^40^K concentrations was also performed in the muscle, lung, liver, and brain tissue samples collected from 10 dead seals found along the Polish coast between 2013 and 2015 (Fig. 1, Table 2).

**Table 2:** Radionuclide activities in the muscle, liver, brain, and lung of the gray seal from the Polish coast

Sampling number	Organ	Date of finding	Weight (kg)	TL* (cm)	^137^Cs (Bq kg ww)	TF (^137^Cs)	^40^K (Bq kg ww)	TF (^40^K)
257	Muscle	26.05.2013	9	104	4.64 ± 2.07	1.35	132.1 ± 54.6	1.16
258	Muscle	28.05.2013	26.2	117.5	5.69 ± 0.47	1.66	77.7 ± 9.8	0.68
267	Muscle	07.06.2013	34.8	123	7.30 ± 0.36	2.13	73.1 ± 6.4	0.64
275	Muscle	17.06.2013	34.8	127.5	5.15 ± 0.52	1.50	88.0 ± 11.8	0.77
327	Muscle	05.05.2014	14	106	8.09 ± 0.46	2.57	82.9 ± 8.8	0.75
328	Muscle	16.05.2014	n.b.	*ca122*	8.10 ± 1.06	2.57	80.7 ± 12.2	0.73
330	Muscle	29.05.2014	9.6	94	6.07 ± 0.76	1.93	78.6 ± 18.3	0.71
340	Muscle	13.03.2015	230.44	225	5.78 ± 0.80	1.95	61.4 ± 8.5	0.56
	Lung	13.03.2015	230.44	225	5.33 ± 0.36	1.79	59.9 ± 7.2	0.54
348	Muscle	13.08.2015	54	160	17.18 ± 0.47	5.78	110.2 ± 5.9	0.99
	Liver	13.08.2015	54	160	9.38 ± 0.39	3.16	76.4 ± 6.5	0.68
	Lung	13.08.2015	54	160	2.59 ± 0.29	0.87	32.5 ± 6.4	0.29
	Brain	13.08.2015	54	160	10.46 ± 0.46	3.52	116.1 ± 8.2	1.04
352	Muscle	22.10.2015	74	170	24.27 ± 0.63	8.17	98.1 ± 6.8	0.88
	Liver	22.10.2015	74	170	18.93 ± 0.47	6.37	77.2 ± 4.8	0.69
	Lung	22.10.2015	74	170	7.52 ± 0.30	2.53	31.2 ± 4.5	0.28
	Brain	22.10.2015	74	170	13.92 ± 1.35	4.69	112.0 ± 6.9	1.00

*TL Length from the tip of the snout to the end of the hind flippers

^137^Cs and ^40^K concentrations were determined in herring (*Clupea harengus*) samples obtained in years 2007–2015 from commercial fishing activities. Twenty to twenty-five specimens were collected at each of four locations (Fig. 1).

### Analytical methods

Until the time of analysis, all samples of placenta and tissue were frozen at − 20 °C. Prior to the analysis, samples were freeze-dried and homogenized. ^137^Cs and ^40^K activity in tissue was measured in the Institute of Meteorology and Water Management—National Research Institute (IMWM) by means of gamma spectrometry method using a HPGe detector with a relative efficiency of 40% and a resolution of 1.8 keV for the 1332 keV peak of ^60^Co, coupled with an 8192-channel computer analyzer and GENIE 2000 software. The measurement time for each sample was 80,000 s. The minimum detectable activities (MDA) stayed at levels of 0.5 Bq kg^−1^ ww and 7 Bq kg^−1^ ww respectively for ^137^Cs and ^40^K. The reference solution, “Standard solution of gamma-emitting isotopes, code BW/Z-62/27/07” produced at the IBJ-Swierk Poland, was used for preparing reference samples for the equipment calibration. The same geometry of cylindrical dishes with a 40-mm diameter (as applied for environmental samples) was used for reference samples during equipment calibration.

Each fish integrated analytical sample was ashed, digested in concentrated nitric acid, and, as a liquid, inserted into vessels of appropriate geometry for radioactivity measurement. The concentrations of ^137^Cs in fish samples were determined in the Central Laboratory for Radiological Protection in Warsaw using a gamma spectrometer with an HPGe detector with an energy resolution of 1.8 keV for ^60^Co (1332 keV), and a relative efficiency of 30%, coupled with a Canberra MULTIPORT II MCA multichannel analyzer with GENIE-2000.

The reliability and accuracy of the measurements were verified annually by the participation of both laboratories in the international intercalibration organized within the HELCOM cooperation. Additionally, IMWM participates in intercomparison exercises on ^137^Cs, ^134^Cs, and ^90^Sr analyses organized yearly by IAEA-MEL Monaco.

Plutonium isotope activities in placenta were measured with the radiochemical method. Plutonium was separated by ion exchange, followed by electrodeposition onto stainless steel disks. Standard reference material 4334H ^242^Pu was used as an internal tracer for counting alpha activity and chemical recovery. Plutonium activity was measured by alpha spectrometry using a PIPS detector with an efficiency of 34% placed in a vacuum chamber (Taipale and Tuomainen 1985). The measurement time for each sample was 500,000 s. The minimum detectable activities (MDA) were equal to 4.04 mBq kg^−1^ ww and 3.11 mBq kg^−1^ ww. The Standard Radionuclide Source Eckert and Ziegler Analytics (EZA) U-238, U-234, Pu-238, and Am-241 were used for preparing reference samples for the equipment calibration. The reliability of the applied method was checked by the determinations of radionuclide concentrations in reference materials (IAEA 465) carried out in 2017.

### Assessment of exposure to radiation

Based on the data on the concentrations of ^137^Cs and ^40^K in individual elements, an attempt was made to estimate the dose rates (taking into account both β and γ radiation) to which the mother is exposed from external and internal sources and the puppy from external sources. To estimate the dose rates, the average values of isotope concentrations in the placenta, muscle, and seawater calculated for each year were used. In addition, in the case of puppies, the final maximum isotope concentrations in bearings representing the maximum exposure of puppies to radiation were adopted. The calculations omit changes in isotope concentrations in the placenta during pregnancy. These changes result from the time of extending the bioaccumulation process, but there may also be a dilution effect associated with an increase in the weight of the placenta during pregnancy. In addition, calculations were made for the minimum and maximum concentration values showing the dose rates range.

In the case of the mother, a dose rate associated with γ and β radiation from the internal source was calculated upon concentrations of ^137^Cs and ^40^K in muscles, assuming they are representative of the whole body. In order to assess the exposure to radiation from external sources, the activity of ^137^Cs and ^40^K in the seawater was taken into account for the mother, while for the pup, the concentrations of isotopes in the placenta were used.

Radiation exposure was assessed for both mother and pup using dose conversion factors derived to Glikov and Brown (2003) and ICPR (2017). In dose rates calculation, the body geometry of seal, 1 × 0.26 × 0.24, was assumed and basing of this the dose coefficients for non-human biota was appointed. For ^137^Cs (combined γ and β), dose coefficients were 0.322 nGy h^−1^ Bq^−1^ kg and 0.147 nGy h^−1^ Bq^−1^ kg respectively for internal and external sources (ICRP 2017). Obtained results were compared with those calculated using separated (for γ and β) dose coefficients: internal γ (1.44E-06 Gy a^−1^ Bq^−1^ kg) and β (1.26E-06 Gy a^−1^ Bq^−1^ kg) and external γ (1.40E-09 Gy a^−1^ Bq^−1^ m^3^) and β (1.06E-13 Gy a^−1^ Bq^−1^ m^3^) (Golikov and Brown 2003). For ^40^K appropriate, dose coefficients were 0.342 nGy h^−1^ Bq^−1^ kg (internal γ and β) and 0.0492 nGy h^−1^ Bq^−1^ kg (external γ and β) (ICRP 2017) and internal γ (3.60E-07 Gy a^−1^ Bq^−1^ kg), internal β (2.64E-06 Gy a^−1^ Bq^−1^ kg), external γ (4.28E-10 Gy a^−1^ Bq^−1^ m^3^), and external β (6.26E-13 Gy a^−1^ Bq^−1^ m^3^) (Golikov and Brown 2003).

## Results and discussion

### Concentrations of radionuclides in the placenta

**Fig. 2:**
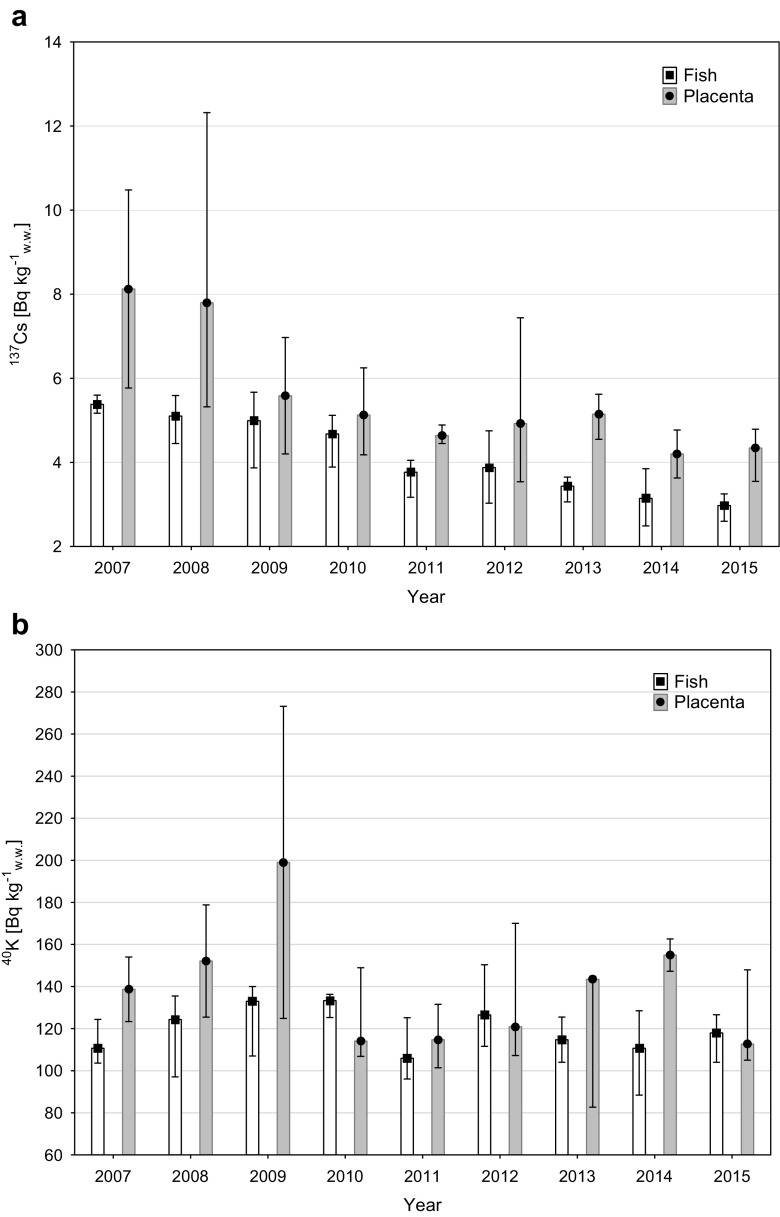
Activity of ^137^Cs (**a**) and ^40^K (**b**) in fish and in the placenta in the 2007–2015 (the bars—average activity and whisker—min-max)

In the years 2007–2015, the activity concentrations of ^137^Cs in seal placenta ranged from 3.5 to 12.3 Bq kg^−1^ ww, while the mean value was 5.5 Bq kg^−1^ ww. These values were significantly lower than those of the potassium isotope—^40^K, which is an isotope of natural origin, but due to the similar properties of K^+^ ions may be competitive in the accumulation process of Cs^+^ ions. The activity concentrations of ^40^K ranged from 82.7 to 273.2 Bq kg^−1^ ww, and the mean value was 136.6 Bq kg^−1^ ww (Table 1). There were no significant differences between the individual mothers, three of whom gave birth 7–8 times during the period, while one of them pupped only twice. The average concentrations of ^137^Cs were respectively 5.5 Bq kg^−1^ ww—seal 1, 6.3 Bq kg^−1^ ww—seal 2, 5.0 Bq kg^−1^ ww—seal 3, and 4.8 Bq kg^−1^ ww—seal 4. In the period from 2007 to 2015, a statistically significant decrease in ^137^Cs concentrations in seal placenta was observed (*r* = − 0.54, *p* = 0.007) (Fig. 2a). In 2007 and 2008, the average concentrations of ^137^Cs remained at level 8 Bq kg^−1^ ww, while in the years 2009–2013, a decrease to approximately 5 Bq kg^−1^ ww was observed. In the last 2 years of study, the average concentrations of ^137^Cs amounted to 4.2 Bq kg^−1^ ww and 4.3 Bq kg^−1^ ww respectively in 2014 and 2015. In the same period, there was a decrease in the concentrations of ^137^Cs in herring caught in the Polish Baltic region, with mean values ranging from 5.4 Bq kg^−1^ ww in 2007 to 3.0 Bq kg^−1^ ww in 2015, which was also statistically significant (*r* = − 0.88, *p* < 0.001) (Fig. 2a). At the same time, it was found that changes in the concentrations of ^137^Cs in fish (herring—*C. harengus*) exhibited a statistically significant correlation (*r* = 0.76, *p* < 0.001) with changes in the concentrations of the discussed isotope in seawater from the same area. The average concentration of ^137^Cs in seawater changed from 42 Bq m^−3^ in 2007 to 19 Bq m^−3^ in 2015, following a trend observed since 1997 which is due largely to radioactive decay and other factors such as bioaccumulation and sedimentation processes, as well as water exchange with the North Sea (Saniewski and Zalewska 2018). In spite of the absence of correlation between ^137^Cs concentrations in the placentas and the concentrations observed in seawater (*p* = 0.078), it was proven that there was a statistically significant correlation between the levels of ^137^Cs in the placenta and those observed in fish that were the staple seal feed (*r* = 0.59, *p* = 0.002). Additionally, the effective half-lives of ^137^Cs calculated for the period 2007 and 2015 were very similar for placenta, herring, and seawater. There were equal to 8.0 years in the southern Baltic Sea seawater, 9.3 years in *C. harengus*, and 8.8 years in the placenta, which support found correlations.

In contrast to the anthropogenic isotope, there was no any trend observed in activities of ^40^K. The average activity of ^40^K in placenta calculated for the whole period was 136.6 Bq kg^−1^ ww, and concentrations were within the range from 82.7 to 273.2 Bq kg^−1^ ww (Fig. 2b).

Concentrations of ^40^K in herring were uniform in the period from 2007 to 2015 and stayed in the range from 106 to 133 Bq kg^−1^ ww (Fig. 2b).

Based on ^137^Cs activity in the particular components of the marine environment, the values of concentration factors (CF) were determined, in this case, expressing the ratio of ^137^Cs concentrations observed in fish to those observed in seawater. Moreover, transfer factors (TF) were calculated as the ratio of the concentrations in a particular seal tissue to the concentrations in fish. Both these factors express the efficiency of bioaccumulation, however, occurring at different levels (Szefer 2002). The calculation of TF values in the case of seals is justified by the fact that the bioaccumulation of heavy metals and radionuclides in larger sea organisms by means of food is a predominant route. The CF values for herring were within the range of 109 to 154 dm^3^ kg^−1^ ww with a mean value of 123 dm^3^ kg^−1^ ww, similar values were determined for the period 2000–2010 (Zalewska and Suplińska 2013). The TF values in placentas remained at a low level, between 0.85 and 2.42, and the average value was 1.34, indicating a small degree of biomagnification in the case of seal placentas. In four cases, the TF was even below 1 (Table 1). The TF values for ^40^K in placentas remained at similar to the isotope of the ^137^Cs level with mean value 1.16 and the range from 0.73 to 2.11. The results of the ^239 + 240^Pu analysis in two placenta samples showed the sum of concentrations of the two isotopes, amounting to 5.0 mBq kg^−1^ ww and 3.3 mBq kg^−1^ ww, to be close to the minimum detectable activity level; therefore, the risk of the presence of these isotopes in the body can be considered negligible.

### Concentrations of radionuclides in other tissue

The average activity of ^137^Cs in the muscle tissue of the dead seals found in the period from 2013 to 2015 was 9.23 Bq kg^−1^ ww (Table 2) and was lower than the mean value (27.0 Bq kg^−1^ ww) determined for the gray seal muscles acquired in 1996 and 1999 (Ciesielski et al. 2015). Higher activity of ^137^Cs was identified in the seal from the Bothnia Bay, 37.4 Bq kg^−1^ ww and 25.4 Bq kg^−1^ ww acquired in 2012 and 2014, respectively (Saremi et al. 2018). The highest ^137^Cs activity concentration of the muscle from seals was 280 Bq kg^−1^ ww for gray seals from the coast of Finland and ringed seals 120 Bq kg^−1^ ww from the Gulf of Finland collected in the short period after Chernobyl accident (Ilus 2007). In 2013, the concentrations of muscles were within the range from 4.6 to 7.3 Bq kg^−1^ ww, while in 2014, they were slightly higher, from 6.1 to 8.1 Bq kg^−1^ ww. The highest activity levels, at 17.2 and 24.3 Bq kg^−1^ ww, were recorded in two specimens found in 2015, which at the same time were markedly larger in terms of length and weight than the specimens found in previous years. Kasamatsu and Ishikawa (1997) reported the ^137^Cs concentration in marine fauna increased with trophic level and demonstrated that in the case of 4 studied marine species (Japanese conger, *Gadus macrocephalus*, *Platycephalus indicus*, *Kareius bicoloratus*), ^137^Cs concentration increased with growth of mass and in the case of 4 species (*Sebastes inermis*, *Girella punctata*, *Hippoglossoides dubius*, *Limanda herzensteini*), no specific correlation was observed. Seals have long life spans (up to 40 years) as consequently that is possible, that in the case of the older (bigger) individuals levels of bioaccumulated radioisotopes are higher. However, based on a small number of results, no statistically significant relationship was found between the size, weight or total length of seals, and ^137^Cs concentrations in their muscles. The authors of other studies have also failed to demonstrate such a correlation (Udaka et al. 2009; Ciesielski et al. 2015; Saremi et al. 2018).

The specimens with the highest levels of ^137^Cs in their muscles (24.3 Bq kg^−1^ ww) also demonstrated relatively high concentrations in other tissues (Table 2). In the case of the liver, ^137^Cs activity was 18.9 Bq kg^−1^ ww, similar to those recorded in 1996 and 1999—17.1 Bq kg^−1^ ww (Ciesielski et al. 2015). In the case of the brain, the obtained results remained on a similar level, 10.5 Bq kg^−1^ ww and 13.9 Bq kg^−1^ ww. The lowest activity of ^137^Cs was determined in the lungs, ranging from 2.5 to 7.5 Bq kg^−1^ ww. Such levels of ^137^Cs concentrations were reflected in TF values determined for individual tissues (Table 2). The average TF value in muscle samples was 2.96 and while in most samples, it remained within a range of 1.35 to 2.57, there were two cases with a three and eightfold increase in ^137^Cs concentrations in the muscle tissue in relation to fish. Also, in the case of the liver (TF from 3.16 to 6.37) and brain (TF from 3.52 to 4.69), the transfer factor indicated visible biomagnification. The lowest TF values (mean 1.73) were determined in the lungs, where they ranged from 0.87 to 2.53. At the same time, it should be emphasized that ^137^Cs concentration values in the tissues of Baltic seals are at much higher levels than in the seals from other regions, as exemplified by ^137^Cs concentrations of 0.25 ± 0.05 Bq kg^−1^fw and 0.12 ± 0.04 Bq kg^−1^fw in the muscle and liver samples of ringed seals in the Arviat area of Canada (Chen et al. 2017). This is most likely the result of the largest contamination of the Baltic Sea waters compared to other areas of the world’s ocean.

The concentrations of ^40^K in muscles varied within a range of 61.4 to 132.1 Bq kg^−1^ ww, while the mean value was 88.3 Bq kg^−1^ ww (Table 2). The concentrations of ^40^K in the two liver samples were at a similar level and were equal to 76.5 Bq kg^−1^ ww and 77.2 Bq kg^−1^ ww, respectively. A ^40^K activity that occurred in the lungs was two times lower (mean value—41.2 Bq kg^−1^ ww) in contrast to the brain, where ^40^K concentrations were at 116 Bq kg^−1^ ww and 112 Bq kg^−1^ ww. TF values calculated for muscle changed in the range from 0.58 to 1.16 giving the mean value 0.79. In the case of the lung and liver, the TF values were definitely below 1, and in the case of the brain, it was equal to 1. The TF values determined for ^40^K indicate lack of enrichment in the case of the examined tissues in the trophic chain.

### Exposure to radiation from internal and external sources

Exposure to radiation from internal sources associated with the levels of ^137^Cs and ^40^K concentrations in the muscle tissue of adult seals was evaluated taking into account both γ and β radiation, emitted by both isotopes. The total dose rates from external and internal sources calculated using conversion factors from the two publications were very similar (Table 3) (Glikov and Brown 2003; ICPR 2017). The average internal dose rates to adult seal associated with γ and β radiation emitted by ^137^Cs were respectively 1.52 nGy h^−1^ and 1.33 nGy h^−1^ and accounted for 42% and 5% of the respective mean dose values associated with the presence of ^40^K (γ—3.6 nGy h^−1^ and β—26.6 nGy h^−1^) (Table 3). Similar relations between internal dose rates associated with γ and β radiation for ^137^Cs and ^40^K were reported by Ciesielski et al. (2015) for harbor propoise and striped dolphin from the southern Baltic Sea. The exposure of adult individuals to radiation from external sources constituted 0.2% of internal dose from ^137^Cs and 0.5% for the potassium isotope compared to doses from internal sources. The mean external radiation dose connected with γ and β radiation emitted by ^137^Cs was 0.005 nGy h^−1^ and 4.09E-07 nGy h^−1^ respectively, which was only 3% and 0.002% of the radiation dose of γ and β from ^40^K (γ—0.16 nGy h^−1^ and β—2.19E-04 nGy h^−1^). Visibly higher radiation doses were estimated in the case of pups, for which the concentrations of ^137^Cs and ^40^K in placentas were assumed as sources of external radiation. The mean radiation dose from ^137^Cs was 0.84 nGy h^−1^ from γ radiation and 6.38E-05 nGy h^−1^ from β radiation, which constituted respectively 12.6% and 0.65% of the γ and β radiation doses from ^40^K (γ—6.64 nGy h^−1^ and β—9.72E-03 nGy h^−1^). The external doses to pup were calculated basing on the isotope concentrations in the placenta after born so when it is the biggest size. Assuming that concentrations of isotopes may increase with pregnancy time, the doses taking the maximal concentrations (at the final stage) can be overestimated. However, in 2013, the average activity of ^137^Cs in two placentas after birth was 5.09 Bq kg^−1^ ww and an average weight of the bearing was 1.85 kg and one after a miscarriage which happened 2 months before the due date was 5.27 Bq kg^−1^ ww with total weight 0.62 kg.

**Table 3:** Calculated internal and external radiation doses in the gray seal (nGy h^−1^)

	Radiation doses				
	Internal γ	Internal β	External γ	External β	
^137^Cs					
Adult	2.97 (1.49–7.81)		0.005 (0.003–0.006)		ICRP 2017
	1.52 (0.76–3.99)	1.33 (0.67–3.490	0.005 (0.003–0.007)	4.09E-07 (2.33E-07–5.34E-07)	Golikov and Brown 2003
Puppy	–		0.77 (0.52–1.81)		ICRP 2017
	–		0.84 (0.566–1.969)	6.38E-05 (4.28E-05–1.49E-04)	Golikov and Brown 2003
^40^K					
Adult	30.19 (20.95–45.18)		0.15 (0.12–0.23)		ICRP 2017
	3.63 (2.52–5.43)	26.60 (18.49–39.82)	0.16 (0.12–0.23)	2.19E-04 (1.74E-04–3.40E-04)	Golikov and Brown 2003
Puppy	–		6.69 (4.07–13.43)		ICRP 2017
	–		6.64 (4.04–13.39)	9.72E-03 (5.91E-03–1.95E-02)	Golikov and Brown 2003

The dose rates from internal and external sources estimated for adults seals would be negligible compared to the doses received from food. Taking into account the average activity concentration ^137^Cs in fish in 2007–2015 (4.1 Bq kg^−1^ ww) and the average fish consumption by seals at the level of 7 kg per day and using available only for human dose coefficient value for ^137^Cs—13 nSv Bq^−1^ and for ^40^K—6.20 nSv/Bq^−1^ (ICRP 2012) dose connected with fish consumption was calculated. The mean annual radiation dose obtained from consumed fish would be 1.93 mSv, of which about 8% comes from ^137^Cs.

## Conclusions

1. The concentrations of ^137^Cs in seal placentas remained within the range from 3.54 to 12.32 Bq kg^−1^ ww, and a statistically significant (*r* = − 0.54, *p* = 0.007) decrease of ^137^Cs concentrations in seal placentas was observed in years from 2007 to 2015.
2. The activity of ^137^Cs in placentas exhibited a statistically significant correlation with the concentration in herring that constituted staple seal food (*r* = 0.59, *p* = 0.002).
3. ^137^Cs concentrations in other tissues were slightly higher than those observed in the placenta; in the case of muscles, they remained in the range from 4.64 to 24.3 Bq kg^−1^ ww. The lowest value was recorded for the lungs—2.59 Bq kg^−1^ ww.
4. ^40^K concentrations in muscles, liver, and lungs varied from 61.4 to 132.1 Bq kg^−1^ ww.
5. The average transfer factor values for ^137^Cs was 1.34 in the placenta, 2.30 in the lung, 2.96 in the muscle, 4.11 in the brain, and 4.77 in the liver.
6. For adults seal, the effective dose from ^137^Cs, from internal (2.97 nGy h^−1^) and external (0.005 nGy h^−1^) sources, constitutes 9.8% of the radiation dose from ^40^K.
7. The mean external radiation dose to pup was 0.77 nGy h^−1^ from ^137^Cs and 6.69 nGy h^−1^ from ^40^K. The share of ^137^Cs in the effective dose is 11.5%, assuming that the only external radiation that a pup is exposed to is associated with the presence of ^137^Cs in the mother’s placenta.

## References

[CR1] Andersen M, Gwynn J, Fuglei E, Dowdall M, Kovacs K, Lydersen C (2006). Radiocaesium (^137^Cs) in marine mammals from Svalbard and the Barents and the Greenland Seas. Sci Total Environ.

[CR2] Carroll J, Wolkers H, Andersen M, Rissanen K (2002). Bioaccumulation of radiocaesium in Arctic seals. Mar Pollut Bull.

[CR3] Ciesielski T, Góral M, Szefer P, Jenssen BM, Bojanowski R (2015). 137Cs 40K and 210Po in marine mammals from the southern Baltic Sea. Mar Pollut Bull.

[CR4] Chen J, Zhang W, Sadi B, Wang X, Muir DCG (2017). Activity concentration measurements of selected radionuclides in seals from Canadian Arctic. J Environ Radioactiv.

[CR5] Golikov V, Brown JE (2003) Internal and external dose models Deliverable Report 4 for EPIC EC Inco-Copernicus Project ICA2-CT-2000-10032 Norwegian Radiation Protection Authority Østerås p 94

[CR6] IAEA (2005) Worldwide marine radioactivity studies (WOMARS). Radionuclide levels in oceans and seas. Final report of a coordinated research project International Atomic Energy Agency 197 pp

[CR7] ICRP (2012) Compendium of dose coefficients based on ICRP Publication 60 ICRP Publication 119 Ann ICRP 41(Suppl) 10.1016/j.icrp.2012.06.038 23025851

[CR8] ICRP (2017) Dose coefficients for nonhuman biota environmentally exposed to radiation ICRP Publication 136 Ann ICRP 46(2) 10.1177/0146645317728022 29205047

[CR9] Ilus E (2007). The Chernobyl accident and the Baltic Sea. Boreal Environ Res.

[CR10] Kasamatsu F, Ishikawa Y (1997). Natural variation of radionuclide 137Cs concentration in marine organisms with special reference to the effect of food habits and trophic level. Mar Ecol Prog Ser.

[CR11] Nehring I, Grajewska A, Falkowska L, Staniszewska M, Pawlicza I, Saniewska D (2017). Transfer of mercury and phenol derivatives across the placenta of Baltic grey seals (Halichoerus grypus grypus). Environ Pollut.

[CR12] Saniewski M, Zalewska T (2016). Atmospheric deposition and riverine load of ^90^Sr and ^137^Cs to the Gulf of Gdańsk (southern Baltic Sea) in the period 2005-2011. J Environ Radioact.

[CR13] Saniewski M, Zalewska T (2018). Budget of 90Sr in the Gulf of Gdańsk (southern Baltic Sea). Oceanologia.

[CR14] Saremi S, Isaksson M, Harding KC (2018). Bio accumulation of radioactive caesium in marine mammals in the Baltic Sea – reconstruction of a historical time series. Sci Total Environ.

[CR15] Szefer P (2002) Metals metalloids and radionuclides in the Baltic Sea ecosystem Volume 5 Elsevier Science 766 pp

[CR16] Taipale TK, Tuomainen K (1985) Radiochemical determination of plutonium and americium from sea-water sediment and biota samples STUK B Valo 26 Helsinki1–Helsink29

[CR17] Udaka M, Ikemoto T, Zenke H, Takahshi S, Batoev VB, Petrov EA, Tanabe S (2009). Radionuclide (^137^Cs and ^40^K) concentrations in the muscle of Baikal seal (Pusa sibirica) from Lake Baikal. Mar Pollut Bull.

[CR18] Zalewska T, Suplińska M (2013). Anthropogenic radionuclides^137Cs^ and ^90^Sr in the southern Baltic Sea ecosystem. Oceanologia.

